# An Iron Rod Pushed through the Abdomen

**Published:** 1859-01

**Authors:** 


### ART. III.—*An Iron Rod pushed through the Abdomen. Recovery.*

BUFFALO, November 29, 1858.

DEAR DOCTOR:

The following account of a penetrating wound of the belly, and recovery, was given me by Mr. A. Knapp, a medical student, and I have sent it to you for publication, as being well worthy of a record.

Yours truly,

FRANK H. HAMILTCN.

In February, 1845, a young man, aged nbout twenty-five, saddle and harness maker by trade, being at work on the first floor, got upon the shop table for the purpose of conversing (through a trap door) with a shoemaker in the room directly overhead; the latter, through sport, motioned to throw a last at the saddler's head, who, in order to avoid the supposed blow had to flex his body very considerably, as his head and shoulders were above the second floor; by so doing he lost his balance and came down from the table in a vertical position, encountering the iron rod used for filling collars, which was four and a half feet in length, three-eights of an inch at the point, slightly flattened and lunated, and some five-eighths at the base, and exceedingly rough without, being newly made by the common smith; the ro passed into the abdomen four inches below the umbilicus, one inch from the linea alba, on the right side, and came out upon the back, on the same side, about opposite the last dorsal vertebra, two inches from the mesian line; he instantly called to the shoemaker, but, pulled out the rod himself before the other got down stairs, walked across the street to his boarding-house, and a surgeon was instantly called, who examined the wounds, and found two drops of blood upon the lining of the waist-band of his pants, which was also pierced. The surgeon immediately closed the wounds with adhesive plaster, directed a low diet, with an occasional enema.

I saw him on the eighth day from the accident. He was sitting up in bed, playing on the violin, in which manner he had been amusing himself for several days. He had suffered no pain, except a slight stinging sensation when he drew out the rod; and he feels no inconvenience, except from hunger and consequent weakness. Subsequently, I saw him at work at his trade, as usual.

This occurred at the Lackawanna Iron Works, in the practice of Dr. Throop, Providence, Luzerne county, Pa.

